# Application of the Horizontal Mattress Techniques in Side-to-Side Microvascular Anastomoses Maximizes Intima Eversion, Technique and Clinical Case Report

**DOI:** 10.1227/neuprac.0000000000000190

**Published:** 2025-12-15

**Authors:** Xiaochun Zhao, Christopher S. Graffeo, Andrew M. Bauer

**Affiliations:** Department of Neurosurgery, University of Oklahoma Health Sciences Center, Oklahoma City, Oklahoma, USA

**Keywords:** Microvascular anastomosis, Side-to-side bypass, Horizontal mattress, Case report

## Abstract

**BACKGROUND AND IMPORTANCE:**

In side-to-side microvascular anastomoses, it's challenging to achieve vessel intima eversion on the back wall. It is sometimes necessary to lengthen the arteriotomy to compensate the elevated risk of thrombosis formation. This note demonstrates application of the horizontal mattress techniques with an illustrative case to maximize intima eversion, which has not been widely adopted in open vascular micro-neurosurgical practice.

**CLINICAL PRESENTATION:**

A 50-year-old female with multiple strokes with transient right sided weakness was diagnosed as bilateral moyamoya syndrome, we present a side-to-side bypass with the horizontal mattress technique. The indocyanine green angiography showed patency of the anastomosis. The patient remained neurologically intact postoperatively. In side-to-side anastomoses, this technique starts with an outside-in (extraluminal-to-intraluminal) stitch at the proximal aspect of the arteriotomy. An aneurysm clip is applied to anchor the suture, the horizontal mattress techniques are subsequently applied to the back wall with suture loops loose, an “outside-in” stitch is followed by an “inside-out” stitch on the contralateral side, and an “inside-out” stitch is followed by an “outside-in” stitch on the ipsilateral side. The suture loops were tightened sequentially proximal-to-distally. A simple tight stitch was then placed at the distal aspect as the inferior anchor, to which the inferior free end of the suture is tightened. Similarly, the proximal free end was tightened after the aneurysm clip is released to complete the backwall.

**CONCLUSION:**

Although technically challenging, the horizontal mattress technique can optimize the intima eversion on the back wall in side-to-side microvascular anastomoses.

The side-to-side anastomosis is a valuable but challenging technique in open neurovascular practice.^1^ Typically, a continuous running technique is required on the back wall given the intraluminal suturing pattern, which leads to elevated thrombosis risk from the edges being inverted thereby potentially bringing the adventitia tissue in contact with the bloodstream.^2^ A longer arteriotomy (2-3 times of vessel diameter) is usually recommended due to this disadvantage.^3^

The horizontal mattress technique has been adopted in microsurgery with improved eversion and better opposition of the intima at the anastomosis rim.^4,5^ The concept of a horizontal mattress suture includes a return “outside-in” stitch on the ipsilateral side when a “inside-out” stitch was performed (Figure 1A), this technique provides optimal intima eversion (Figure 1B).

**FIGURE 1.**
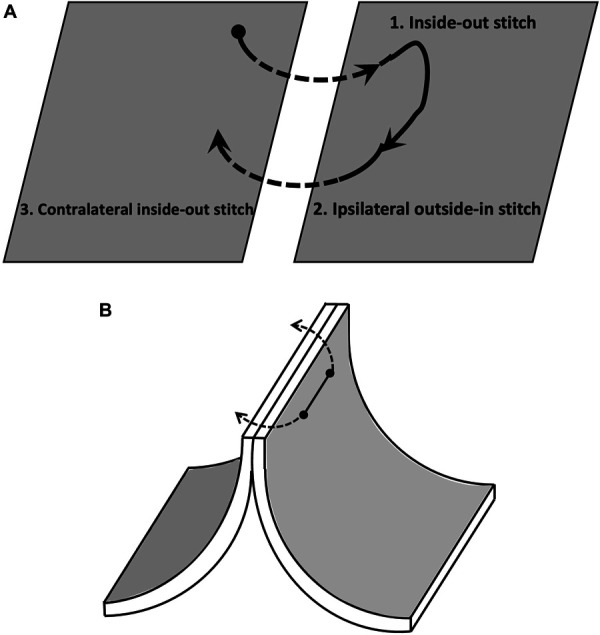
Concept of the horizontal mattress technique: **A**, Principles of the horizontal mattress, an “inside-out” stitch is always followed by an ipsilateral “outside-in” stitch and an outside-in stitch is always followed by a contralateral “inside-out” stitch. **B**, The nature of horizontal mattress technique maximizes intima eversion.

This technical note aims to apply the continuous horizontal mattress techniques to the back wall in a side-to-side anastomosis to maximize intima eversion. We present technique demonstration on vessel simulation tubes as well as an illustrative case.

## CLINICAL PRESENTATION

**FIGURE 2.**
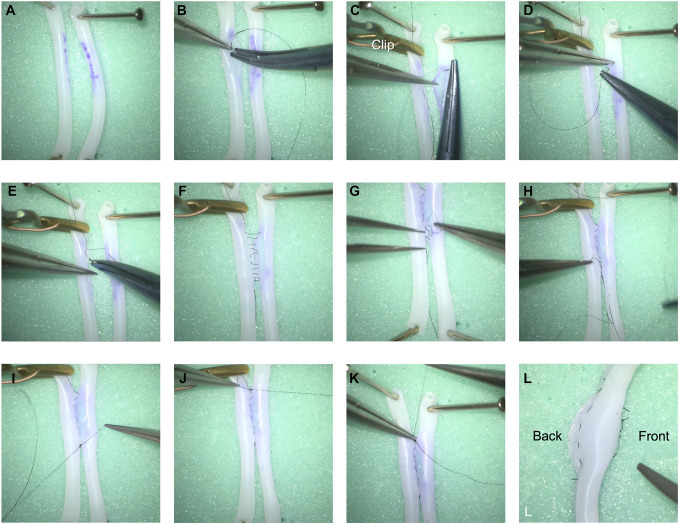
Demonstration of steps in a side-to-side bypass using the horizontal mattress technique. **A**, Two simulation tubes are positioned side by side with gently inferiorly curved arteriotomy marked with purple dye. **B**, The arteriotomies are performed and the initial stitch is placed at superior end on the left vessel as an “outside-in” stitch. **C**, An aneurysm clip is placed to anchor the superior thread end to the left vessel, and an “inside-out” stitch is placed on the right (contralateral) vessel. **D**, Following the “inside-out” stitch on the right vessel, an “outside-in” stitch was thrown in on the right (ipsilateral) side. **E**, An “inside-out” stitch is then placed on the left (contralateral) side. **F**, Following this simplified principle, the entire back wall is sutured with loose loops with the continuous horizontal mattress technique. **G**, The loose thread loops are tightened sequentially superior-inferiorly with the anchoring clip in place. **H**, A simple stitch was placed at the inferior end of the arteriotomy as the inferior anchor. **I**, The inferior end of the continuous horizontal mattress thread is tied to the inferior anchor after the loops are tightened. **J**, A simple stitch was placed at the superior end of the arteriotomy as the superior anchor and the clip can be removed. **K**, The superior end of the thread is then tied to the new superior suture anchor after confirming the entire back wall was tightened. **L**, The front wall is then closed with simple interrupted technique, as a comparison, the optimal intima eversion is demonstrated on the back wall.

**FIGURE 3.**
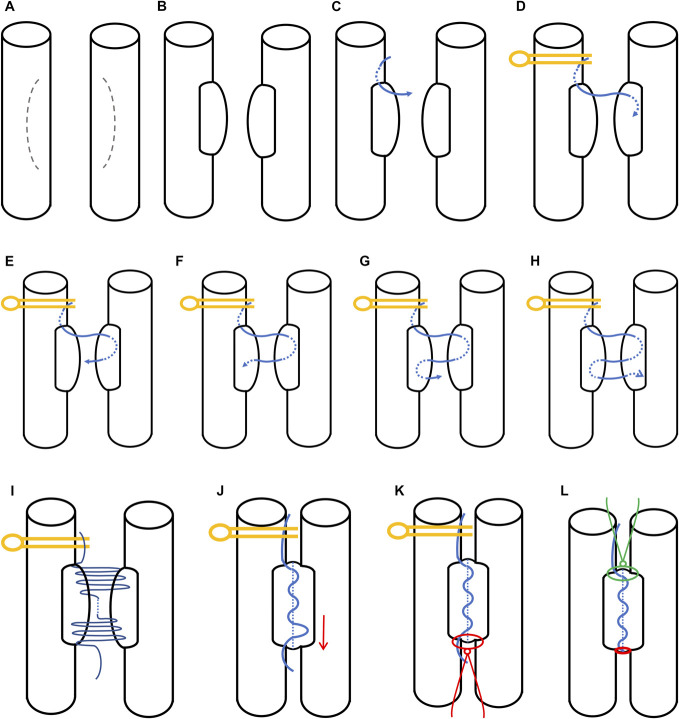
Artistic illustrations of the horizontal mattress technique: **A**, The 2 vessels are positioned parallel with the arteriotomy planned with a gentle curve based inferiorly. **B**, The arteriotomy was opened on both vessels with the curved flap providing extra tissue on the back wall. **C**, The first stitch is placed superiorly on the left vessel in an “outside-in” fashion. **D**, An aneurysm clip is placed superior to the entry of the initial stitch as an anchor point; and an “inside-out” stitch is placed on the contralateral (right) side. **E**, An “outside-in” stitch is placed on the ipsilateral side (right) side. **F**, Subsequently, an “inside-out” stitch is placed on the contralateral (left) side. **G**, Then a return “outside-in” stitch is placed on the ipsilateral (left) side. **H**, Following the same principle, an “inside-out” stitch is placed on the contralateral (right) side. **I**, A series of loose thread loops form after completion of the back wall. **J**, With the clip anchoring superiorly, the loops are tightened superior-inferiorly. **K**, A regular suture was placed at the inferior end of the arteriotomy, which the free thread end is tied to. **L**, Another simple stitch is placed superiorly after removing the clip and the superior thread is secured to it. This completes the back wall with the horizontal mattress techniques.

Two 2 mm tubes were placed side by side to mimic an in situ side-to-side anastomosis (Figure 2A), the arteriotomies were planned with gently curved flaps pointing inferiorly (Figures 2A, 3A and 3B, Video 1).

The first stitch starts from the left vessel on the high end, which is a “outside-in” stitch (Figures 2B and 3C), based on the principle of the horizontal mattress technique, an “inside-out” stitch is placed on the contralateral vessel wall (Figures 2C and 3D), at this time an aneurysm clip is placed to anchor the superior free thread to secure the set-up and provide tension in the next steps; this “inside-out” stitch is then followed by an “outside-in” stitch on the ipsilateral wall (Figures 2D and 3E); the new “outside-in” stitch then becomes the new start stitch, following the same principle, an “inside-out” stitch is placed to the contralateral side (Figures 2E and 3F). Every stitch should be placed loose with no tightening that there is room to place the next stitch with good visualization and maneuverability. Continuing the suturing pattern, the back wall can be finished with loose thread (Figures 2F and 3G-3I).

The loose thread loops are subsequently tightened proximally to distally with the anchoring clip in place providing sufficient tension (Figures 2G and 3J), a regular simple stitch was placed at the inferior end of the arteriotomy, to which the inferior free thread end was tied to, after the thread was tightened and secured (Figures 2H, 2I and 3K). With the inferior end secured, the clip can be removed, and a regular stitch was placed at the superior end of the arteriotomy, and the superior free thread end was secured to it (Figures 2J, 2K and 3L). This concludes the back wall horizontal mattress technique. The front wall can be completed in any technique given the somewhat lower risk of vessel inversion, here we demonstrated a simple interrupted technique in the figure (Figure 2L), as comparison, 1 could appreciate the excellent intima eversion on the back wall.

### Illustrative Case

A 50-year-old female presents with multiple strokes with transient right sided weakness over the period of 6 months, related to hypoperfusion secondary to bilateral moyamoya syndrome, which was confirmed with diagnostic angiogram (Video 2). She underwent right sided superficial temporal artery to middle cerebral artery anastomoses using the “mini-max” technique,^6^ with a side-to-side anastomosis to a temporal branch and an end-to-side anastomosis to a frontal branch. The above-mentioned horizontal mattress technique was applied to the back wall of the side-to-side anastomosis, and the simple running technique was carried out on the front wall. The indocyanine green angiography demonstrated patency of the anastomosis with good intima eversion of the back wall. The total occlusion time of the side-to-side anastomosis was 54 minutes. She remained neurologically intact postoperatively and was discharged next day. The patient provided consent to the surgical procedure and publication of this surgical technique, Institutional review board approval was not required given the de-identified nature of patient information.

## DISCUSSION

Although technically challenging, the horizontal mattress technique can be a potential solution to the suboptimal intimal eversion in current techniques in side-to-side anastomosis.

The curved arteriotomy can provide greater prominent bulge, this was advocated by Tanikawa et al,^1^ however this is applied to the front surface while the back wall was closed by traditional running suture, to reduce the risk of thrombosis mainly by expanding the lumen. Here, we recommend the bulge to be on the back wall (C shaped arteriotomy with the base facing down, Figure 3A) to maximize the chance of intima eversion. By nature, the horizontal mattress technique will cause lumen stenosis to certain degree, the C shaped design with a flap could provide more tissue to achieve optimal intima eversion on the back wall (at highest risk) while minimizing sacrificing the lumen caliber.

The application of continuous horizontal mattress on the back wall can be somewhat counterintuitive as the intima is facing up to the surgeon while the outside surface is facing down. However, this can be simplified following some basic principles: any “outside-in” stitch will be followed by an “inside-out” stitch on the contralateral vessel wall, and any “inside-out” stitch will be followed by an “outside-in” stitch on the ipsilateral vessel wall. The clip could provide temporary fixation of the superior thread while the loops are individually tightened and secured inferiorly; as the 2 sides of the backwall need to be separated loose while the horizontal mattress technique is being applied to have visualization.

In this case, usually the “inside-out” stitch will require switching the needle direction (can be back-handed) to place the “outside-in” stitch on the ipsilateral wall, this can be time consuming and requires decent amount of microsurgical practice. The vessel occlusion time was 54 minutes in our illustrative case, which is significantly longer than a traditional approach, this is the major disadvantage of this technique. With practice and improvement of the needle handling (change of needle direction), there could be potential to reduce some time. On the other hand, the arteriotomy may not need as long of a segment as in the traditional techniques, this could decrease some occlusion time in future practice.

For the front wall, we demonstrated both traditional technique and horizontal mattress technique as comparison, which elucidate the optimal eversion with intima-to-intima contact favoring the horizontal mattress technique. However, any traditional technique (interrupted or continuous) can accomplish the front wall suturing given that care is taken to evert the edges on the back wall. With practice and reduced time, the horizontal mattress technique can be applied in any type of anastomosis to optimize eversion and reduce thrombosis risk.

## CONCLUSION

In conclusion, the horizontal mattress technique can be applied to the backwall in a side-to-side anastomosis, with optimal intima eversion to reduce thrombosis risk. We present the first illustrative case of application of this technique in a superficial temporal artery to middle cerebral artery anastomosis. Application of this technique can be challenging; however, excellent results can be achieved following basic principles and microsurgical training.

## References

[1] Gomez-Vega JC Ota N Kusdiansah M Noda K Kamiyama H Tanikawa R. A practical guide to train the side-to-side anastomosis: tips, tricks and technical nuances. World Neurosurg. 2024;189:17-25. 38750884 10.1016/j.wneu.2024.05.034

[2] Acland RD Russell RC. Practice manual for microvascular surgery. Plast Reconstr Surg. 1990;85(3):475.

[3] Wang L Cai L Qian H Lawton MT Shi X. The in situ side-to-side bypass technique: a comprehensive review of the technical characteristics, current anastomosis approaches, and surgical experience. World Neurosurg. 2018;115:357-372. 29729474 10.1016/j.wneu.2018.04.173

[4] Orak I Güneren E Yildiz L. A new technique for microvascular anastomosis: eversion with 3 horizontal mattress sutures. Ann Plast Surg. 2006;57(1):80-83. 16799314 10.1097/01.sap.0000203999.59856.c5

[5] Başar H Erol B Tetik C. Use of continuous horizontal mattress suture technique in end-to-side microsurgical anastomosis. Hand Surg. 2012;17(3):419-427. 23061959 10.1142/S0218810412970064

[6] Arnone GD Hage ZA Charbel FT. Single vessel double anastomosis for flow augmentation–a novel technique for direct extracranial to intracranial bypass surgery. Oper Neurosurg. 2019;17(4):365-375. 30690506 10.1093/ons/opy396

